# Vaccination with a bacterial peptide conjugated to SARS-CoV-2 receptor-binding domain accelerates immunity and protects against COVID-19

**DOI:** 10.1016/j.isci.2022.104719

**Published:** 2022-07-05

**Authors:** Athanasios Blanas, Haiko Karsjens, Aafke de Ligt, Elisabeth J.M. Huijbers, Karlijn van Loon, Stepan S. Denisov, Canan Durukan, Diederik J.M. Engbersen, Jan Groen, Sven Hennig, Tilman M. Hackeng, Judy R. van Beijnum, Arjan W. Griffioen

**Affiliations:** 1 Angiogenesis Laboratory, Department of Medical Oncology, Cancer Center Amsterdam, Amsterdam UMC, Amsterdam, the Netherlands; 2 School for Cardiovascular Sciences, Department of Biochemistry, Maastricht University, Maastricht, the Netherlands; 3 Department of Chemistry & Pharmaceutical Sciences, Amsterdam Institute of Molecules, Medicines and Systems, Vrije Universiteit Amsterdam, Amsterdam, the Netherlands; 4 CimCure BV, The Hague, the Netherlands; 5 Intravacc, Institute for Translational Vaccinology, Bilthoven, the Netherlands

**Keywords:** Biological sciences, Immunology, Immune response, Virology

## Abstract

Poor immunogenicity of critical epitopes can hamper vaccine efficacy. To boost immune recognition of non- or low-immunogenic antigens, we developed a vaccine platform based on the conjugation of a target protein to a chimeric designer peptide (CDP) of bacterial origin. Here, we exploited this immune Boost (iBoost) technology to enhance the immune response against the receptor-binding domain (RBD) of the SARS-CoV-2 spike glycoprotein. Despite its fundamental role during viral infection, RBD is only moderately immunogenic. Immunization studies in mice showed that the conjugation of CDP to RBD induced superior immune responses compared to RBD alone. CDP-RBD elicited cross-reactive antibodies against the variants of concern Delta and Omicron. Furthermore, hamsters vaccinated with CDP-RBD developed potent neutralizing antibody responses and were fully protected from lung lesion formation upon challenge with SARS-CoV-2. In sum, we show that the iBoost conjugate vaccine technology provides a valuable tool for both quantitatively and qualitatively enhancing anti-viral immunity.

## Highlights

• An iBoost-based CDP-RBD conjugate vaccine against SARS-CoV-2
• Induction of potent RBD-specific humoral and cellular responses
• CDP-RBD vaccination protects hamsters from lung lesion formation

## Graphical abstract

**Figure:**
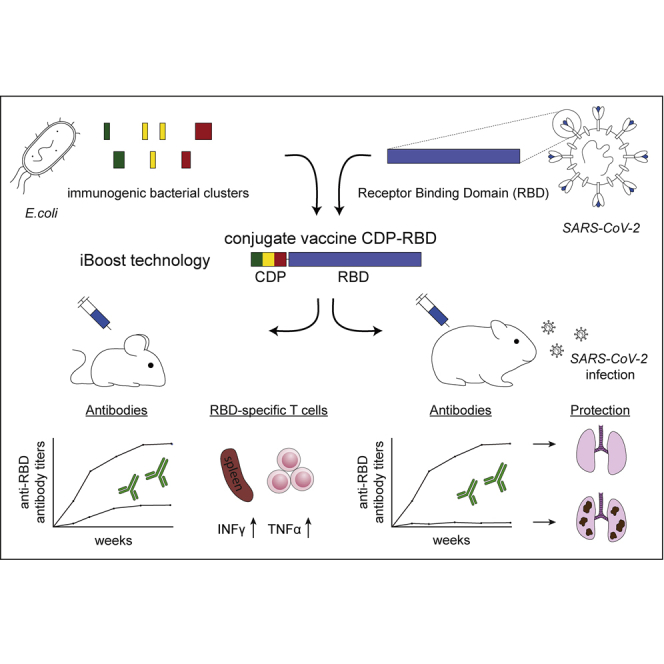

## Introduction

The rapid spread of severe acute respiratory syndrome coronavirus 2 (SARS-CoV-2) across the globe has led to more than 500 million confirmed infections and at least 6 million registered deaths until June 2022 (https://covid19.who.int/). SARS-CoV-2 is responsible for the coronavirus disease 2019 (COVID-19), which is associated with systemic pathology, excessive lung inflammation, and significant mortality (Huang et al., 2020; Li et al., 2020; Smadja et al., 2021). With the currently applied vaccination programs, it seems possible to successfully fight the pandemic. However, major challenges remain. For example, the continuously evolving SARS-CoV-2 mutated versions, such as the Delta and Omicron variants (Abdool Karim and de Oliveira, 2021; Callaway, 2021) keep menacing the effectiveness of the approved vaccines and the longevity of protection may not be as long as expected. These issues potentially keep society, healthcare systems, and economies at continued risk worldwide. Therefore, novel vaccine platforms that pave the way for the development of updated, effective, faster acting, and safe vaccine regimens are still evidently needed.

The vast majority of neutralizing antibodies detected in the serum of patients with COVID-19 are directed against the receptor-binding domain (RBD) (Wrapp et al., 2020) of the SARS-CoV-2 spike protein S (Barnes et al., 2020; Cho et al., 2021; Ortega et al., 2021). RBD is responsible for cell entry and subsequent infection through binding to angiotensin-converting enzyme 2 (ACE-2) expressed by host cells (Barnes et al., 2020; Zhou et al., 2020). Antibodies targeting RBD offer significant protection against the development of severe disease (Wang et al., 2020; Wu et al., 2020). However, it has been suggested that RBD is moderately immunogenic by itself (Nanishi et al., 2022; Sun et al., 2021; Tan et al., 2021). Hence, induction of a robust immune response against this critical domain of the SARS-CoV-2 spike protein by vaccination is of utmost importance for limiting new infections and preventing high numbers of hospitalization.

Several RBD-based vaccines using different technologies (DNA, mRNA, viral vector, nanoparticle) have been designed and tested in preclinical and clinical settings (Kleanthous et al., 2021). However, these vaccine platforms are characterized by considerable limitations in terms of storage conditions, global production capacity, and scalability (Koff et al., 2021). In contrast, the production of protein-based subunit vaccines is established for many decades and is easily scalable and more affordable. One drawback of the protein subunit vaccines is that their efficacy is greatly dependent on the solubility and the intrinsic immunogenicity of the target antigen (Vartak and Sucheck, 2016). Thus, to concentrate the immune response towards soluble RBD and enhance its immunogenicity in the context of an RBD-based subunit vaccine, we investigated the possibility of using the conjugate vaccine technology that we have previously developed for the induction of antibody responses against self-antigens overexpressed in tumors (Huijbers and Griffioen, 2017; Huijbers et al., 2018, 2019). This vaccine platform, called immune Boost (iBoost), aims at increasing targeted immune recognition of non- or low-immunogenic epitopes by conjugating them to an engineered chimeric designer peptide (CDP) sequence of bacterial origin. We hypothesized that the application of the iBoost technology for RBD-targeted vaccination against SARS-CoV-2 can successfully protect against COVID-19.

Our results demonstrate that the iBoost-based CDP-RBD conjugate vaccine is capable of inducing superior, i.e. faster, stronger, and more mature humoral and cellular responses in mice compared to its unconjugated counterpart. Furthermore, induced antibodies show cross-reactivity against variants of concern (VOC) such as Delta and Omicron. In addition, this approach elicits effective neutralizing antibody responses in hamsters and offers protection against lung lesion formation after SARS-CoV-2 infection. Importantly, the described technology holds promise for future vaccination programs against other viruses or infectious pathogens.

## Results

### Production of an iBoost-based receptor-binding domain-targeting conjugate vaccine

**Figure 1:**
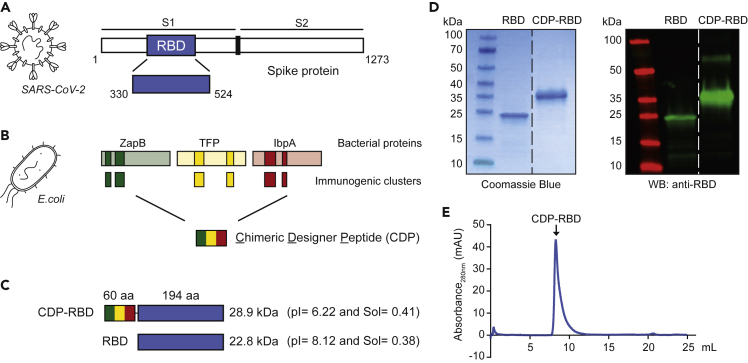
A conjugate vaccine targeting the RBD domain of SARS-CoV-2 (A–C) Schematic representation of the RBD (A), CDP (B), and produced CDP-RBD (28.9 kDa) and RBD (22.8 kDa) vaccine proteins (C). (B) CDP is composed of several highly immunogenic clusters originating from three different bacterial proteins (cell division protein ZapB, type-1 fimbrial protein TFP, and small heat shock protein IbpA). (C) The theoretical isoelectric point (pI) and the predicted solubility score (Sol) for each vaccine protein. (D) Coomassie staining after SDS-PAGE (left) and anti-RBD Western blot (right) analysis showing RBD and CDP-RBD. (E) Size-exclusion chromatogram (10/300 GL Superdex 75, Cytiva) showing purified CDP-RBD (black arrow).

To achieve robust immune recognition of the RBD of SARS-CoV-2 (Figure 1A), its sequence was conjugated to CDP (Figure 1B). CDP consists of selected clusters of amino acids with bulky hydrophilic or charged side chains, originating from three distinct bacterial (*E. coli*) proteins, the cell division protein ZapB (UniProtKB: P0AF36), the type I fimbrial protein (A chain) (TFP, UniProtKB: P04128) and the small heat shock protein IbpA (UniProtKB: P0C054). The resulting conjugate protein CDP-RBD, as well as RBD alone (Figure 1C), were expressed in BL21 bacteria (*E. coli* strain) and subsequently purified as previously described (Huijbers et al., 2018). Notably, the addition of the CDP component to RBD improved the solubility of the final conjugate protein (Figure 1C) and increased the protein yield (Figures S1A and S1B). Purified proteins were validated by SDS-PAGE and Western blot analysis using a commercial anti-RBD antibody (Figure 1D). Also, size exclusion chromatography confirmed the purity (area under the curve of the peak equates to 90%) of CDP-RBD (Figure 1E). Lastly, *in silico* analysis of the CDP-RBD protein sequence revealed the α-helix or β-strand domains, as well as the predicted B cell- and CD4^+^/CD8^+^ T cell epitopes (Figure S1C), suggesting potent immune recognition.

### Chimeric designer peptide-receptor-binding domain induces faster and stronger antibody responses compared to receptor-binding domain alone

**Figure 2:**
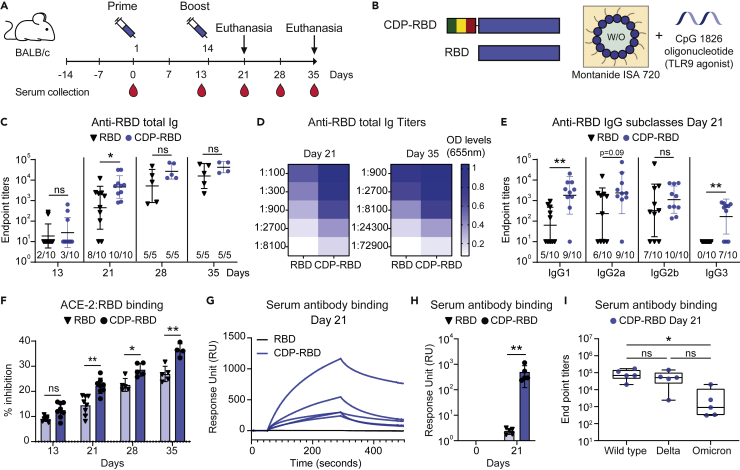
A conjugate CDP-RBD vaccine elicits faster antibody responses compared to its unconjugated counterpart (A) Timeline of mouse vaccinations. Mice were immunized with 100 μg of RBD or CDP-RBD on days 0 and 14. Sera were collected from all mice before vaccination (day 0) and at experimental days 13, 21, 28, and 35 (n = 10 for days 13 and 21, n = 5 for days 28 and 35). (B) Vaccine proteins were mixed with Montanide ISA 720 together with CpG 1826 oligonucleotide (MnC). (C) Anti-RBD total immunoglobulin (Ig) endpoint titers for both vaccine groups at experimental days 13, 21, 28 and 35 as assessed by ELISA. The number of responding mice is provided for each timepoint. (D) Anti-RBD total immunoglobulin (Ig) titers at day 21 (left) and day 35 (right). (E) Analysis of endpoint titers of RBD-specific IgG subclasses (IgG1, IgG2a, IgG2b, IgG3) at day 21. The number of responding mice is provided for each IgG subclass. (F) Surrogate virus neutralization assay. Circulating neutralizing antibodies in the sera of vaccinated mice inhibit the interaction between RBD and the ACE-2 receptor. (G and H) Surface Plasmon Resonance (SPR) biosensor assay. Binding of anti-RBD antibodies of mouse sera toward commercial RBD. Sera were diluted 1:100. (I) Total immunoglobulin (Ig) endpoint titers of CDP-RBD immunized mice at experimental day 21 against the wild type-RBM, Delta-RBM and Omicron-RBM were assessed by ELISA. Sera were diluted 1:100. Data are shown as geometric mean values ± geometric SD (C and E), as mean values ±SD (D, F, and H) or as Box-and-whisker plots (I). Statistical significance was determined by an unpaired, Mann-Whitney test for each time point (C and H) or IgG subclass (E) or by a two-way ANOVA followed by Sidak’s multiple-comparison test (F and I). (∗p < 0.05, ∗∗p < 0.01).

To assess the immunogenicity of CDP-RBD *in vivo* and compare it to its unconjugated RBD counterpart, BALB/c mice were immunized subcutaneously with 100 μg of each purified protein by a prime vaccination on day 0 and a booster vaccination on day 14 (Figure 2A). Based on its previously reported safety and efficacy, Montanide ISA 720 (water-in-oil emulsion) supplemented with a CpG 1826 oligonucleotide (Toll-like receptor 9 agonist) was selected as a vaccine adjuvant (referred to as MnC) (Figure 2B) (Huijbers et al., 2012). Murine sera were collected before immunization (day 0), as well as on days 13, 21, 28, and 35 (Figure 2A) and were used for the ELISA detection of anti-RBD antibodies. No antibodies against the RBD domain were present in the pre-immune sera (day 0). Seroconversion was detectable by day 13 in a couple of mice from each group (Figures 2C and S2A) and by day 21 all mice immunized with CDP-RBD had developed a strong immunoglobulin (Ig) response against RBD. At this time point, only 50% of the RBD-immunized mice displayed a potent RBD-specific humoral response (Figures 2C, 2D left, and S2A), while no anti-RBD antibodies were detected in 2/10 mice receiving the unconjugated RBD vaccine (Figure 2C). This result confirmed the moderate immunogenicity of the RBD. Importantly, although the difference in anti-RBD total Ig levels among the two vaccine groups seemed to disappear at later time points (Figure S2A), evaluation of the antibody titers revealed that RBD-immunized mice were still lagging behind their CDP-RBD counterparts even three weeks after the booster injection (Figures 2C and 2D right). These findings illustrate that using the iBoost platform for vaccination against RBD results in not only faster, but also stronger anti-RBD antibody responses relative to RBD alone.

Next, we sought to investigate the production of IgG subclasses. IgG1 and IgG2b are representative of T helper (Th)2-skewed immune responses, whereas IgG2a and IgG3 associate with the induction of a Th1 response (Weber et al., 2014). By day 21, immunization of mice with CDP-RBD resulted in significantly higher levels of both anti-RBD IgG1 and IgG3 relative to vaccination with RBD (Figures 2E and S2B). In addition, there was a trend towards a more robust and homogeneous IgG2a response in the CDP-RBD group (Figures 2E and S2B). Interestingly, upon immunization with CDP-RBD, but not with unconjugated RBD, a higher number of mice were capable of mounting a complete IgG1, IgG2a, IgG2b, and IgG3 response (Figure 2E). For example, RBD-specific IgG3 was measured in 7/10 CDP-RBD-immunized mice, whereas none of the mice injected with RBD were found IgG3-positive (Figures 2E and S2B). Hence, the CDP-RBD vaccine ensures a more complete response (based on the presence of all IgG subtypes), as early as seven days after the second vaccination (day 21).

Given the vigorous immune recognition of RBD accomplished after conjugation to CDP, we assumed that CDP-RBD recipient mice also develop anti-CDP antibodies. On day 21, CDP-specific antibodies were detected only in the CDP-RBD group (Figure S2C). Taking into consideration the improved RBD-specific antibody response observed in mice immunized with the CDP-RBD conjugate vaccine, immune reaction to the bacterial CDP fusion partner did not seem to negatively influence the immunogenicity of the RBD antigen. Moreover, to exclude the possibility that the differences between the two vaccine groups are dependent on the adjuvant used, rather than the presence of CDP specifically, we immunized mice with RBD or CDP-RBD in combination with the alternative adjuvant Sepivac (Sep) in an identical experiment (Figure 2A). In contrast to Montanide, which is a water-in-oil emulsion, Sepivac is an oil-in-water adjuvant that is currently being tested for several influenza and COVID-19 vaccines. Again, CDP-RBD/Sep outperformed the RBD/Sep vaccine in terms of anti-RBD total Ig induction (Figure S2D), in a similar manner as CDP-RBD/MnC, indicating that CDP is the key component responsible for boosting the humoral immune response. Importantly, CDP-RBD combined either with MnC or Sep was capable of inducing potent antibody responses against RBD even at lower protein amounts than 100 μg (Figure S2E). In combination with Sepivac, CDP-RBD induces a potent immune response even at a protein concentration of 10 μg (Figure S2E). A surrogate neutralization assay revealed that sera derived from CDP-RBD-immunized mice displayed higher inhibition of RBD:ACE-2 binding compared to sera from RBD-immunized mice from day 21 onwards (Figure 2F). This underscores the superior neutralization capacity obtained already one week after the second vaccination with the conjugate CDP-RBD vaccine. In line with this, surface plasmon resonance (SPR) analysis revealed that day 21 sera of mice vaccinated with CDP-RBD exhibited a significantly higher level of binding towards immobilized RBD than sera from RBD-vaccinated mice (Figures 2G and 2H). Interestingly, we observed similar results with the Sepivac adjuvant (Figures S2G and S2H) and the differences among the RBD and CDP-RBD groups were largely maintained until day 35 (Figures S2I and S2J), indicating that the conjugate vaccine technology significantly improves affinity maturation and leads to enhanced antibody avidity towards RBD (Bauer, 2021).

Recent VOC display increased number of mutations in the RBD, more specifically in the receptor-binding motif (RBM). This subdomain of RBD contains all the residues directly interacting with the ACE-2 receptor expressed on the surface of host cells (Lan et al., 2020), and thus, it is the major target of neutralizing antibodies (Niu et al., 2021). For this reason, we were also interested in testing whether CDP-RBD vaccination induces cross-reactive antibodies against the RBM of Delta and Omicron VOC. Interestingly, we observed reactivity against the RBM-Omicron, although it was lower compared to the reactivity against the RBM with the original sequence (RBM-wild type) and RBM-Delta (Figure 2I). This finding is in line with other studies, which show a reduced reactivity of serum antibodies from convalescent and vaccinated individuals against Omicron, but not against the Delta variant (Gattinger et al., 2022; Rossler et al., 2022).

### Systemic T cell immunity and enhanced CD8^+^ T cell responses are induced upon vaccination with chimeric designer peptide-receptor-binding domain

**Figure 3:**
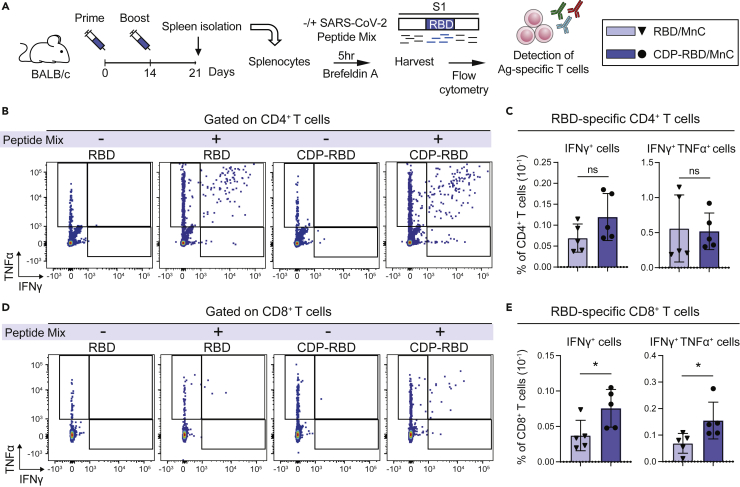
CDP-RBD vaccination evokes systemic T cell immunity and further improves the CTL response in mice compared to vaccination with RBD (A) Timeline and experimental procedure followed for the detection of splenic RBD-specific T cells in immunized mice. (B and D) Representative dot plots depicting IFNγ and TNFα expression in CD4^+^ T cells (B) and CD8^+^ T cells (D) upon no stimulation (−) or *ex vivo* stimulation (+) with a SARS-CoV-2 Peptide Mix for 5 h in the presence of Brefeldin A. (C and E) Quantification of the percentage (%) of RBD-reactive IFNγ^+^ (monofunctional) and IFNγ^+^ TNFα^+^ (polyfunctional) CD4^+^ (C) and CD8^+^ (E) T cells on vaccination with the RBD/MnC or CDP-RBD/MnC regimens (n = 5 mice per group). Each dot represents the mean value of two technical replicates. Error bars indicate mean frequencies ±SD. Statistical significance was determined by an unpaired Student’s t test (ns; no significance, ∗p < 0.05).

Besides antibodies, antigen-specific T cells are also crucial for the clearance of SARS-CoV-2 infection (Nelde et al., 2021). Thus, we next aimed to determine whether CDP-RBD induces stronger T cell responses in mice compared to unconjugated RBD. To assess this, the spleens of vaccinated mice were excised on day 21, as this was the point in time where the two vaccine groups started differing in their antibody responses and neutralization capacity. Splenocytes were restimulated *ex vivo* with a pool of 15 amino acid-long peptides covering the S1 domain (which includes RBD) (Aiello et al., 2021; Chan et al., 2021; Thieme et al., 2020) of the spike protein (Figure 3A). Non-stimulated splenocytes were taken along as a negative control. After stimulation for 5 h in the presence of Brefeldin A, the frequency of antigen-specific CD4^+^ and CD8^+^ T cells was examined using flow cytometry (Figure S3A), based on the induction of interferon-gamma (IFNγ) and tumor necrosis factor-alpha (TNFα) expression (Figures 3B-3D). More specifically, we were able to identify a clear population of monofunctional (IFNγ^+^ or TNFα^+^), as well as multifunctional (IFNγ^+^ TNFα^+^) (Seder et al., 2008) CD4^+^ T cells (Figures 3B, 3C, and S3B), but without major discernible differences among the two vaccine groups. On the contrary, a significantly higher percentage of both monofunctional and multifunctional RBD-specific CD8^+^ T cells was detected in the spleens of CDP-RBD-immunized mice compared to their RBD-immunized counterparts (Figures 3D, 3E, and S3B), suggesting that conjugation to CDP has a positive impact on the systemic cytotoxic T cell (CTL) response. To further clarify the link between the humoral and cellular immune responses in these vaccine groups, we tested the correlation between the anti-RBD antibodies and the RBD-specific T cells (Figures S3C–S3E). Interestingly, multiple components of the antibody response were found to be positively correlated with the T cell response in CDP-RBD-immunized mice (except for polyfunctional CD8^+^ T cells), while IgG1 was the predominant IgG subtype showing high correlation with T cells in the case of RBD recipients. Taken together, these data suggest that the conjugate technology facilitates the induction of a coordinated and fine-tuned antibody and T cell response very quickly after the second vaccine dose.

### The chimeric designer peptide-receptor-binding domain vaccine protects against COVID-19 in hamsters

**Figure 4:**
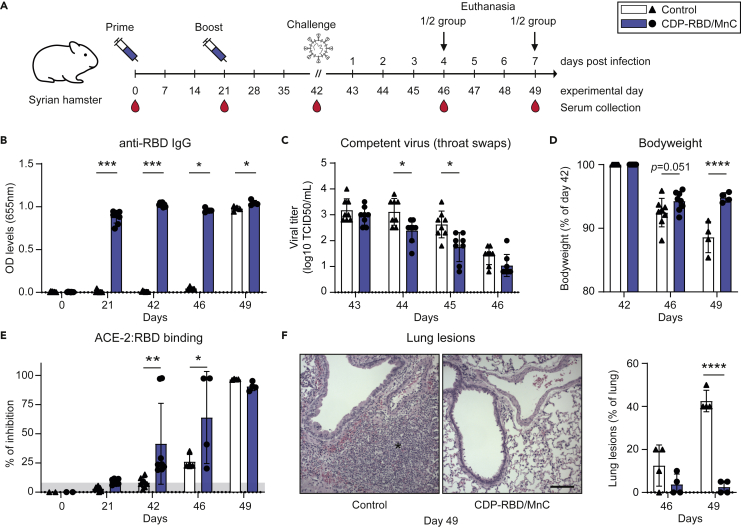
CDP-RBD elicits neutralizing antibodies and protects against SARS-CoV-2-mediated lung lesions in hamsters (A) Timeline of the vaccination strategy and SARS-CoV-2 challenge of hamsters. Animals were immunized with 100 μg of CDP-RBD protein in combination with MnC as adjuvant or with Tris-sucrose (control) at day 0 and day 21. Sera were collected from all hamsters on experimental days 0, 21, 42, 46, and 49. On day 42 all hamsters were challenged with SARS-CoV-2. Four days post-infection (dpi) half of the animals per group (4 out of 8) were sacrificed, at day 49 (7 dpi) the four remaining animals were sacrificed. (B) Anti-RBD IgG levels at experimental days 0, 21, 42, 46 and 49 as assessed by ELISA. Sera were diluted 1:100. (C) Throat swaps were analyzed to measure the replication of competent SARS-CoV-2 the first four dpi. (D) Weight progression after infection with SARS-CoV-2. At day 49 only the remaining 4 hamsters were weighed. (E) Surrogate virus neutralization assay with sera of hamsters on experimental days 0, 21, 42, 46, and 49. (F) Hematoxylin and eosin (HE) stained images of infected hamster lungs seven dpi (left). Lungs of control hamsters show strong infiltration of immune cells (asterisk). Quantification of lung lesions four and seven dpi (right). Scale bar, 100 μm. Data are shown as mean values ±SD. Statistical significance was determined by an unpaired, Mann-Whitney test for each time point (A) or two-way ANOVA followed by Sidak’s multiple-comparison test (C-F). (∗p < 0.05, ∗∗p < 0.01, ∗∗∗p < 0.001, ∗∗∗∗p < 0.0001).

We further investigated whether the CDP-RBD vaccine, besides inducing an RBD-specific antibody and T cell response, also offers protection against SARS-CoV-2 infection. Therefore, we immunized Syrian hamsters intramuscularly with CDP-RBD/MnC or Tris-sucrose only (referred to control) at days 0 and 21. Three weeks after the booster vaccination (experimental day 42), all hamsters were challenged intranasally with 10^4^ SARS-CoV-2 virus (wild type) particles, which is equal to the median tissue culture infectious dose (TCID50), and serum samples were taken four and seven days post-infection (dpi) (Figure 4A). We found that the CDP-RBD vaccine elicited high antibody titers against RBD after a single vaccine dose (day 21), while the booster vaccination increased the anti-RBD antibody titers further (Figures 4B and S4A). As expected, control hamsters showed no anti-RBD antibodies after vaccination. However, seven days after the challenge with SARS-CoV-2, control hamsters also displayed high anti-RBD antibodies, although these did not reach the antibody titers detected in the CDP-RBD vaccinated hamsters (Figures 4B and S4A). In line with the higher antibody titers, CDP-RBD vaccinated hamsters showed a faster clearance of the virus from the throat. More precisely, at two and three dpi significantly lower amounts of replicating virus were found (Figure 4C). Moreover, lower amounts of virus were also found in the lungs, as well as in the nasal turbine, of CDP-RBD vaccinated hamsters at four dpi (Figure S4B). After SARS-CoV-2 infection, both groups lost around 5% of their initial bodyweight in the first four dpi. Nevertheless, CDP-RBD vaccinated hamsters maintained their bodyweight from this time point on (day 46), whereas control vaccinated hamsters lost significantly more weight (Figure 4D). To elucidate whether CDP-RBD-induced antibodies are characterized by neutralizing capabilities, a surrogate neutralization assay was performed (Figure 4E). Antibodies of CDP-RBD-vaccinated animals hampered the binding of RBD to ACE-2 already on the day of viral challenge (day 42). Significantly improved neutralization compared to control group continued at four dpi. One week after viral challenge (day 49), hamsters of both groups exhibited high inhibition of binding between RBD and ACE-2 (Figure 4E). Strikingly, histopathological analyses performed on lung tissues showed typical COVID-19 characteristics such as congestion, emphysema, hemorrhage, bronchioloalveolar hyperplasia and inflammation or edema in all control hamsters, but not in CDP-RBD vaccinated hamsters. Up to 40% of the lung tissue showed lesions in control hamsters seven dpi, whereas CDP-RBD vaccinated hamsters presented no evidence of SARS-CoV-2 pathology (Figure 4F). This finding is comparable with mRNA and vectored DNA vaccines that have been tested in a similar hamster challenge model (Fischer et al., 2021; Meyer et al., 2021; van der Lubbe et al., 2021). Overall, our data prove that CDP-RBD vaccination induces a potent immune response and protects against severe pathological changes in the lungs induced by SARS-CoV-2 infection.

## Discussion

Here, we present the idea of boosting the immunogenicity of a viral antigen by conjugating it to a bacterial chimeric sequence in the context of a protein-based subunit vaccine. The strategy of enhancing immune recognition of epitopes that display weak immunogenicity by linking them to a bacterial antigen was presented nearly 100 years ago (Avery and Goebel, 1929). On this basis, we recently developed the iBoost technology for improved vaccination against self-antigens, which are non-immunogenic owing to peripheral tolerance (Huijbers et al., 2018). A critical component of the iBoost technology is CDP (chimeric designer peptide), which consists of bacterial protein sequences enriched in highly immunogenic amino acid clusters. We hypothesized that CDP could also prove beneficial for improving immune responses against critical, but only moderately immunogenic viral sequences, such as the RBD domain of the SARS-CoV-2 spike protein. Therefore, we conjugated CDP to the RBD sequence and this resulted in significantly improved immune recognition of the RBD antigen.

We observed that the immunization of mice with the conjugate CDP-RBD vaccine results in faster, stronger, and more balanced IgG responses, as compared to vaccination with unconjugated RBD. Importantly, CDP-RBD vaccination induces RBD-specific antibody titers of 10^4^ magnitude, which are directly comparable to the titers presented in other studies independently of the vaccine technology (DNA-, mRNA-, nanoparticle-based) used each time (Kleanthous et al., 2021). Moreover, similar antibody titers against RBD have been observed in vaccinated or COVID-19 convalescent individuals (Mulligan et al., 2020; Sahin et al., 2020). Induction of an early immune response is considered of utmost importance during targeting highly infectious agents, such as SARS-CoV-2, especially in a pandemic situation. Additionally, it has been shown that mature antibody responses reduce the risk of COVID-19 mortality (Pavel et al., 2021). Here, we observed an improved IgG response in the CDP-RBD group, which was also more homogeneous (less variation in titers among the recipients) and more complete (efficient induction of all IgG subtypes) compared to the RBD group. Notably, only CDP-RBD-immunized mice were able to produce IgG3, an IgG subclass that is associated with complement activation (Collins, 2016). Neutralizing antibodies are considered an established correlate of protection against COVID-19 (Cromer et al., 2022). In relation to this, we found that the combination of CDP with RBD can significantly increase serum neutralization capacity compared to RBD alone in mice from day 21 onwards and this difference is maintained until day 35. Most of the studies assessing antibody neutralization with the surrogate virus neutralization test are using 1/5 or 1/10 serum dilutions. In these cases, the final neutralization score exceeds 50% and is approximately 80-90%. However, in our study, owing to the limited volume of sera derived from mice vaccinated with RBD or CDP-RBD, we were only able to use 1/20 serum dilutions. Therefore, we believe that this is a suboptimal selected dilution and it could explain the final neutralization score (∼40%) that we observed. At the same time, using SPR analysis, we found that CDP-RBD-derived serum antibodies bind significantly more to immobilized RBD, which is indicative of better affinity maturation, higher avidity, and consistent with the improved inhibition of ACE-2:RBD binding. The difference between the RBD-specific antibody levels detected with ELISA and the low cumulative signal from the SPR analysis in the RBD group could be explained by the fact that the SPR signal does not necessarily follow a linear concentration curve. As the sera from both RBD/MnC and RBD/Sep groups exhibited very low binding potential to immobilized RBD compared to their CDP-RBD counterparts both at day 21 and day 35, despite the presence of RBD-specific antibodies, we conclude that our conjugate technology significantly boosts the overall quality of the elicited antibody response. Furthermore, CDP-RBD-elicited antibodies showed cross-reactivity against the RBM subdomain of the Omicron VOC, albeit to a smaller extent compared to the reactivity against RBM-Delta. This could be explained by the fact that, compared to the Delta variant, a higher number of mutations is found within the RBD domain of the Omicron virus, resulting in increased immune escape and reduced antibody reactivity (Hu et al., 2022), which would probably lead to lower virus neutralization.

Besides humoral immunity, the induction of cellular immune responses is essential for protection against viruses (Rouse and Sehrawat, 2010), especially as it has been shown that antibodies wane soon after infection or vaccination (Chvatal-Medina et al., 2021). Along with the numbers, the functionality of SARS-CoV-2-specific T cells determines the timing and the efficiency of virus clearance from the body and it is inversely correlated with disease severity (Le Bert et al., 2020). Furthermore, certain SARS-CoV-2 VOCs are unable to escape recognition by S-specific memory T cells, as it often happens with antibodies (Geers et al., 2021). For this reason, special attention is given to the generation of SARS-CoV-2-specific T cells upon vaccination. An established method for the *ex vivo* assessment of antigen-reactive T cells either in mice (Zhuang et al., 2021) or humans (Le Bert et al., 2020; Wang et al., 2021) includes the quantification of cells expressing the Th1 cytokines IFNγ and TNFα after restimulation with a pool of overlapping peptides corresponding to the target antigen. In this study, CDP-RBD-immunized mice exhibited both CD4^+^ and CD8^+^ RBD-specific T cells in their spleen, indicative of successful induction of a systemic cellular response one week after the booster vaccination. Despite the differences in the total Ig response, we observed a similar frequency of RBD-specific CD4^+^ T cells among the two groups. Nevertheless, compared to immunization with unconjugated RBD, CDP-RBD increased the frequency of reactive monofunctional and multifunctional CD8^+^ T cells, which are supported by Th1 cells and exert a pivotal role in killing infected cells on encounter of their cognate antigen (Schmidt and Varga, 2018; van Lier et al., 2003). This suggests that the use of the iBoost technology can improve the underlying cytotoxic T cell responses and thus, increase protection in vaccinated individuals, as CD8^+^ T cells specific for certain coronavirus epitopes correlate with the development of mild COVID-19 (Schmidt and Varga, 2018). We also noticed that the proportion of splenic RBD-specific CD8^+^ T cells was smaller than the one of the CD4^+^ T cells. Previous reports have indicated that the memory response in the context of SARS-CoV-2 infection is skewed more toward the CD4^+^ rather than the CD8^+^ T cell compartment (Grifoni et al., 2020; Habel et al., 2020). Besides infection, though, vaccine-induced CD8^+^ T cell responses are also significantly lower than their CD4^+^ counterparts. For example, results from a phase-I clinical trial focusing on vaccination with mRNA-1273 (Moderna) revealed that the IFNg^+^ CD4^+^ response dominated over the induced IFNg^+^ CD8^+^ T cell response (Jackson et al., 2020). In addition, vaccines developed by other companies were unable to trigger prominent CD8^+^ T cell responses (Kleanthous et al., 2021). Therefore, our findings are in full agreement with these observations and confirm a less potent induction of antigen-specific CD8^+^ T cells compared to CD4^+^ T cells on vaccination. Finally, CDP-RBD-immunized mice displayed a better correlation of the antibody response to the cellular response compared to their RBD counterparts. This suggests that the presentation of a viral antigen in the context of a conjugate vaccine with a selected foreign partner has the potential to mobilize both arms of the adaptive immune response in a more robust and well-orchestrated manner than that of the viral antigen alone.

Apart from the improved immune response in mice, we also demonstrated that CDP-RBD protects against severe COVID-19 in a hamster model. CDP-RBD-vaccinated hamsters showed strong antibody responses and no lung lesions, as well as decreased weight loss, compared to controls after the SARS-CoV-2 challenge. The protection against lung pathologies is comparable to already approved vaccines tested in the hamster challenge model (Fischer et al., 2021; Meyer et al., 2021; van der Lubbe et al., 2021; Wu et al., 2021). Protection was achieved despite the minor ∼0.5 log10 TCID50 reduction of competent virus in the throat (per mL) and nasal turbines (per gram tissue). However, CDP-RBD vaccinated animals showed ∼3 log10 TCID50/g reduction of competent virus in the lungs four dpi, suggesting that the protection of lung lesions is strongly dependent on the viral load in the lungs rather than in the upper respiratory tract. This observation is in line with SARS-CoV-2 infection in humans, where the replication of the virus in the lower airways is the main cause of increased mortality (Sulaiman et al., 2021). Furthermore, a decrease of ∼3 log10 TCID50/g in the lungs is comparable to the reduction of specific VOCs after vaccination with the approved ChAdOx1 nCoV-19 (AZD1222) vaccine (Fischer et al., 2021). In line with this observation, the two animals which showed no signs of lung lesions at experimental day 46 are the same animals that showed a strong reduction of competent virus in the lungs (∼5 log10 TCID50/g) and high levels of neutralizing antibodies (∼100% inhibition of ACE-2:RBD binding). CDP-RBD-stimulated neutralizing antibody levels were comparable with those on natural infection one week after the challenge. Although natural infection resulted in high neutralizing antibody levels after seven days, moderate neutralization was already achieved three weeks after the second vaccination/the day of viral challenge in CDP-RBD-immunized hamsters. Furthermore, significantly improved neutralization capabilities were observed four dpi, underlining the importance of an early manifestation of neutralizing antibodies for viral clearance and patient survival (Dispinseri et al., 2021).

In general, protein subunit vaccines are a well-established technology with proven efficacy and safety profiles for many years, inter alia, pneumococcal polysaccharide, or MenACWY vaccines (Rappuoli et al., 2019). Moreover, protein vaccines are relatively stable at a refrigerator-friendly 2-8°C range, compared to mRNA vaccines. Therefore, it is easier to distribute them and more economic to implement them, especially in countries with limited resources. The iBoost platform does not only induce faster and stronger B- and T cell immune responses but it also allows specific targeting of critical epitopes within key viral proteins (such as the receptor-binding domain of the spike protein). Furthermore, iBoost offers the possibility to display domains, regardless of their original immunogenicity, of different viral variants (e.g. the Delta and Omicron SARS-CoV-2 variants) or even conserved sequences (e.g. among SARS-CoV-1, -2, and MERS-CoV). This way, iBoost can facilitate the design and further development of multi-epitope vaccines that induce a more targeted immune response and thereby might offer protection against new circulating variants (Cohen et al., 2021). In sum, the iBoost platform represents a promising approach for future vaccination strategies against different viruses and pathogenic microorganisms.

### Limitations of the study

We have used BL21 bacteria (*E. coli*) for the production of the RBD and CDP-RBD proteins. In general, prokaryotic expression systems facilitate the fast production of high protein yields at a very low cost (Porowinska et al., 2013). However, one important disadvantage is the lack of post-translational modifications (e.g glycosylation) in the produced proteins. Although there are two *N*-glycosylation sites (N331, N343) in the RBD sequence (Zhao et al., 2021), previous studies have indicated that RBD produced in BL21 bacteria largely maintains its secondary and tertiary structure and remains completely functional (strong binding to the ACE-2 receptor) (He et al., 2021; Maffei et al., 2021). In line with this, we hereby show that vaccination with CDP-RBD produced in BL21 bacteria induces potent antibody and T cell responses against RBD and ensures the protection of hamsters from lung lesion formation after SARS-CoV-2 infection. Nevertheless, we recognize that the use of a mammalian expression system (e.g HEK293 cells) in the context of other target protein antigens, displaying higher complexity in terms of glycosylation compared to RBD, could be favorable and combined with the proposed iBoost technology.

## STAR★Methods

### Key resources table

**Table:** 

REAGENT or RESOURCE	SOURCE	IDENTIFIER
**Bacterial and virus strains**		

BL21 (DE3)	Sigma-Aldrich	Cat#: 69450
TOP10	Invitrogen	Cat#: C404010
SARS-CoV-2	European Virus Archive Global	BetaCoV/Munich/BavPat1/2020

**Recombinant DNA**		

CDP-RBD plasmid	Genscript	Lot#: U462LFI150-2/TG557138
RBD plasmid	Genscript	Lot#: U462LFI150-6/K60678

**Chemicals, peptides, and recombinant proteins**		

Isopropyl-β-D-thiogalactopyranoside (IPTG)	Serva	26600.04
Ethylenediaminetetraacetic acid disodium salt dehydrate (EDTA)	Sigma-Aldrich	E5134
N-Lauroylsarcosine sodium salt	Sigma-Aldrich	Cat#: L5125
Phenylmethanesulfonyl fluoride solution (PMSF)	Sigma-Aldrich	Cat#: 93482
Urea, 99.5%, for analysis	Acros Organics	Cat#: 10665572
NaCl	VWR	Cat#: 27788.366
NaH2PO4	Merck	Cat#: 1.06346.1000
Phosphate Buffer Saline (PBS)	VWR	Cat#: L0500-500
Ni-NTA Agarose	Qiagen	Cat#: 30210
100GR Imidazole BAKER ANALYZED Reagent	JT Baker	Cat#: 1747.0100
Glass Microfiber Filters/Grade 13400	Sartorius	Cat#: 13400-100------K
Mini-PROTEAN TGX Gels	Bio-rad	Cat#: 4561094 / 4561096
Immobilon-FL PVDF Membrane	Millipore	Cat#: IPFL00010
TWEEN® 20	Sigma-Aldrich	Cat#: P7949
Bovine Serum Albumin Fraction V	Roche	Cat#: 10735086001
Imidazole	J.T.Baker	Cat#: 1747
3,3′,5,5′-Tetramethylbenzidine (TMB) Liquid Substrate System for ELISA	Sigma-Aldrich	Cat#: T0440
16% PFA	Electron Microscopy Sciences	Cat#: 15710
Saponin, pract., from Quillaja Saponaria Molina	Acros Organics	Cat#: 419231000
SARS-CoV-2 spike protein (RBD, His & Avi tag)	Genscript	Cat#: Z03483
PepTivator® SARS-CoV-2 Prot_S1	Miltenyi Biotec	Cat#: 130-127-041
Spectra™ Multicolor Broad Range Protein Ladder	Thermo Fisher Scientific	Cat#: 26634
Coomassie Blue	Serva	Cat#: 17524.02
RPMI 1640 Medium	Biowest	Cat#: L0495-500
New Born Calf Serum	Biowest	Cat#: S0750
Penicillin-Streptomycin (10,000 U/mL)	Thermo Fisher Scientific	Cat#: 15140-122
2-mercaptoethanol	Gibco	Cat#: 31350-010
Brefeldin A Solution (1,000X)	BioLegend	Cat#: 420601
SEPIVAC SWE	Seppic	Cat#: 1849101 Lot: L00621
Montanide ISA 720	Seppic	Cat#: 36059VFL2R3 Lot: 2314805
CpG oligo	Eurogentec	Cat#: 1826 Lot: 2314805

**Antibodies**		

SARS-CoV-2 spike RBD Antibody, Monoclonal Mouse IgG2B Clone # 1034522	R&D systems	Cat#: MAB10540 Lot: CNDN0120083
Polyclonal Goat Anti-Mouse Immunoglobulins/Biotinylated	Dako	Cat#: E0433 Lot: 20065645/20072921; RRID: AB_2687905
Goat Anti-Mouse IgG1, Human ads-BIOT	Southern Biotech	Cat#: 1070-08 Lot: C0716-V3170; RRID: AB_2794413
Goat Anti-Mouse IgG2a, Human ads-BIOT	Southern Biotech	Cat#: 1080-08 Lot: J4016-PA57B; RRID: AB_2794479
Goat Anti-Mouse IgG2b, Human ads-BIOT	Southern Biotech	Cat#: 1090-08 Lot: A2413-R488C; RRID: AB_2794523
Goat Anti-Mouse IgG3, Human ads-BIOT	Southern Biotech	Cat#: 1100-08 Lot: K2613-V9172; RRID: AB_2794575
Goat Anti-Mouse IgM, Human ads-BIOT	Southern Biotech	Cat#: 1020-08 Lot: K2915-N9070; RRID: AB_2737411
Goat Anti-Mouse IgA-BIOT	Southern Biotech	Cat#: 1040-08 Lot: J4416-VM28D; RRID: AB_2794374
Streptavidin/HRP	Dako	Cat#: P0397 Lot: 20059909/20071980
Goat Anti-Hamster IgG(H + L)-BIOT	Southern Biotech	Cat#: 6060-08 Lot: K146-X127H; RRID: AB_2796131
TruStain FcX™ (anti-mouse CD16/32) Antibody, Clone: 93	BioLegend	Cat#: 101320 Lot: B318396; RRID: AB_1574975
GK1.5 (AF700) [anti-mouse CD4]	BioLegend	Cat#: 100429 Lot: B313084; RRID: AB_493698
YTS156.7.7 (AF488) [anti-mouse CD8b]	BioLegend	Cat#: 126627 Lot: B321690; RRID: AB_2800618
XMG1.2 (APC) [anti- mouse IFN-γ]	BioLegend	Cat#: 505809 Lot: B335091; RRID: AB_315403
MP6-XT22 (PE) [anti-mouse TNFα]	BioLegend	Cat#: 506305 Lot: B327716; RRID: AB_315426
Zombie Aqua™ Fixable Viability Kit	BioLegend	Cat#: 423101 Lot: B331243
Goat anti mouse IRDye 800CW	LI-COR	Cat#: 926-32210 Lot: C91210-09; RRID: AB_621842

**Other**		

Clear Flat-Bottom Immuno Nonsterile 96-Well Plates	Thermo Fisher Scientific	Cat#: 442404
Plaat, 96w, F-bodem, PS, 382μL/w, 127,8x85,6mm	Greiner Bio-One	Cat#: 655101
96-well V-bottom plate	Cellstar	Cat#: 651180
Fisherbrand™ Sterile Cell Strainers, Mesh size: 40μm	Fisher Scientific	Cat#: 11587522
Thermoshake incubator shaker	Gerhardt	https://www.gerhardt.de
Soniprep 150 Ultrasonic Disintegrator	MSE	N/A
Odyssey Infrared Imaging System (Model 9120)	LI-COR	https://www.licor.com
Synergy HT Plate Reader	BIO-TEK	https://www.biotek.com
LSRII (Fortessa)	BD Biosciences	

**Critical commercial assays**		

Plasmid isolation Midi kit	Qiagen	Cat#: 12143
Micro BCA™ Protein Assay Kit	Thermo Scientific	Cat#: 23235

**Software and algorithms**		

Prism version 8	GraphPad	https://www.graphpad.com
FlowJo version 10	TreeStar	https://www.flowjo.com

### Resource availability

#### Lead contact

Further information and requests for resources should be directed to and will be fulfilled by the Lead contact, Arjan W. Griffioen (a.griffioen@amsterdamumc.nl).

#### Materials availability

The vaccines produced in this study are available upon request.

### Experimental model and subject details

#### *E. coli* strains for protein production

Competent Top10 *E coli* bacteria were used to make RBD and CDP-RBD plasmids. Competent BL21 (DE3) *E. coli* bacteria were used to express RBD and CDP-RBD proteins.

#### Mice used for vaccination study

BALB/c OlaHsd wildtype mice were purchased from Envigo. Young female mice (17–23 g, 8 weeks old) were used throughout all experiments. All mouse experiments were approved by the Dutch national ethics board Centrale Commissie Dierproeven (CCD, registration number AVD11400202010545) and were performed in agreement with Dutch guidelines and law on animal experimentation.

#### Hamsters used for SARS-CoV-2 challenge study

The challenge study was performed together with Intravacc BV (Netherlands) and subcontracted to Viroclinics Biosciences B.V. (Netherlands). Male Syrian hamsters (*Mesocricetus auratus*, 98–114 g, 9 weeks old) were purchased from Janvier (France).

### Method details

#### Vaccine design

The generation of the CDP fusion partner was described before (Huijbers et al., 2018). Briefly, the genome of *E. coli* (*E. coli*) (strain K12) was examined and three proteins highly enriched in clusters of amino acids with hydrophilic or charged side chains were identified: type-1 fimbrial protein TFP, cell division protein ZapB and the small heat shock protein IbpA. These immunogenic clusters were fused in order to form one single peptide, named chimeric designer peptide (CDP), with a total length of 60 amino acids (5.8 kDa). For the RBD, the sequence encoding amino acids 330 until 524 (in total 194 amino acids) of the reported SARS-CoV-2 genome (GenBank: MN908947.3) was used. Additionally, a (GS)_3_-linker (GSGSGS) between the CDP and RBD domain, as well as a terminal His_6_-tag (HHHHHH), were added to facilitate proper antigen display and vaccine purification, respectively.

#### Vaccine protein production

The DNA sequences encoding RBD or CDP-RBD (codon optimized for expression in *E. coli*) were synthesized by Genscript (USA) and inserted between the Nde1 and Xho1 restriction sites of a pET21a (+) expression vector. For protein expression, 10 ng plasmid DNA was transfected by the heat shock method into competent BL21 (DE3) *E. coli* bacteria (Sigma-Aldrich, #69450). A single clone was cultured overnight in LB-medium supplemented with ampicillin (100 μg/mL) in a non-humified shaker (Laboshake) at 200 rpm and 37 °C. 1mM Isopropyl β-*d*-1-thiogalactopyranoside (IPTG) (Serva, #26600.04) was added to a 1:2.6-diluted overnight culture to induce protein expression. After 4 h, bacteria were harvested and bacterial pellets (equivalent to 50mL culture volume) were dissolved in 5 mL sonication buffer, containing 0.5 M EDTA (Sigma-Aldrich, #E5134), 1% N-lauroylsarcosine (Sigma-Aldrich, #L5125), 1% Phenylmethanesulfonyl fluoride solution (PMSF) (Sigma-Aldrich, #93482) and 6 M Urea (Acros Organics, #10665572) in PBS (VWR, #L0500-500). Proteins were released from the bacteria by high frequency vibration (sonication) for 15 cycles of 20 s “on” and 30 s “off” on ice (Soniprep 150 MSE, amplitude 22–24 microns). For protein purification, the supernatants (after centrifugation at 4500 rpm for 20 min) of sonicated samples were mixed with 200 μL 50% Ni-NTA agarose slurry (Qiagen, #30210) and incubated on a roller bench overnight at 4⁰C. The next day, Ni-NTA agarose beads were washed five times with wash buffer, containing 1 M NaCl (VWR, #27788.366) and 0.05% Tween-20 (Sigma-Aldrich, #P7949) in PBS. Subsequently, beads were transferred to a column, a syringe with a glass filter (Sartorius, #13400-100------K). Proteins were eluted in two steps: first four fractions of 100 μL each were eluted in 1 M Tris-Cl (pH 8.0), 100 mM NaCl, 200 mM imidazole (JT Baker, #1747.0100), 1 mM PMSF and secondly four fractions of 100 μL each in 10 mM Tris-Cl (pH 4.5), 100 mM NaH_2_PO_4_ (Merck, #1.06346.1000), 8 M urea. CDP-RBD was eluted in all eight fractions, whereas RBD was eluted only in the last four fractions containing 8 M urea. Fractions of purified proteins were pooled and stepwise dialyzed (4 M, 3 M, 2.5 M, 2 M urea) against 2 M urea. Final protein concentration was determined by BCA assay (Thermo Scientific, #23235). SDS-PAGE, Coomassie blue staining (Coomassie® Brilliant Blue G 250, Serva, #17524.02), western blot analysis, and size exclusion chromatography were performed to confirm purity and identity of the proteins.

#### Western Blot

Identity of the proteins was confirmed by western blot. Approximately 20 μg of purified protein was loaded on Mini-PROTEAN® TGX™ precast protein gels (Biorad, #4561094, 4561096) and gel electrophoresis was performed. Subsequently, proteins were transferred to an immobilon PVDF membrane (Millipore, #IPFL00010). Membranes were blocked with PBS containing 0.05% Tween-20 (PBS-T) and 5% BSA fraction V (Roche, #10735086001) for 1 h at room temperature. Rabbit anti-RBD antibody (R&D systems, #MAB10540, 1/250 dilution) was used as primary antibody overnight at 4⁰C. The next day, 5 washes with 0.05% PBS-T were performed before adding the secondary antibody, goat anti-mouse IRDye 800CW (LI-COR Biosciences, #926-32210, 1/10000 dilution), for 30 min to the membranes. After five washes with PBS-T and one wash with PBS, proteins were visualized with an Odyssey Infrared Imaging System (Model 9120, LI-COR Biosciences). Prestained spectra multicolor broad range protein ladder (Thermo Fisher, 26634) was used as size reference.

#### Analytical gel filtration assay

Analytical gel filtration was conducted using the ÄKTA Pure system equipped with a Superdex 75 10/300 GL column (Cytiva) in 2 M urea in phosphate-buffered saline (PBS, pH 7.4) at a flow rate of 0.5 mL/min. Each injection contained 100 μL sample with a concentration of 2 g L^−1^. The absorbance was monitored at 280 nm.

#### Prediction of B, CD4^+^ and CD8^+^ T cell epitopes

Linear B cell epitopes in the CDP-RBD sequence were predicted by BepiPred (Jespersen et al., 2017) applying an epitope threshold of 0.55. For the prediction of potential CD4^+^ T cell epitopes we exploited the NetMHCIIpan 4.0 server (Reynisson et al., 2020). Specifically, the affinity of all the overlapping 15 amino-acid long peptides included in the CDP-RBD sequence was tested for binding to the murine H-2 alleles (H-2-IAu, H-2-IEd, H-2-IEk), as well as to several common human HLA-DP, HLA-DQ and HLA-DR alleles. Similarly, we identified predicted CD8^+^ T cells epitopes of 8 amino-acid length in CDP-RBD using the NetMHCpan 4.1 server (Jurtz et al., 2017). The binding to the murine H-2 alleles (H-2-Db, H-2-Dd, H-2-Dq, H-2-Kb, H-2-Kd, H-2-Kk, H-2-Kq, H-2-Ld, H-2-Lq) and different human HLA class I alleles was examined.

#### Mouse vaccinations

BALB/c OlaHsd mice (Envigo) were used for two independent immunization studies (n = 5 per group each). One study was terminated at day 21 and the other study at day 35. After acclimatization for 2 weeks, mice received a prime vaccination on day 0 and a booster vaccination on day 14 (Figure 2A). Each mouse was injected subcutaneously in the left groin with 100 μg of purified CDP-RBD or RBD, respectively. Proteins were mixed 40:50:10 with Montanide ISA 720 (Seppic, #36059VFL2R3) and 50 μg CpG oligo 1826 (Eurogentec, #1826), abbreviated as MnC. For the concentration study, every mouse was injected subcutaneously in the left groin with either 100, 30, 10 or 3 μg of purified RBD or CDP-RBD in combination with Montanide ISA 720 and CpG (40:50:10, MnC) or Sepivac SWE (Seppic, #1849101, 50:50), respectively. Blood samples were collected from the tail vein prior to immunization (day 0) and on days 13, 21, 28 and 35. One mouse from the CDP-RBD MnC group was sacrificed between day 28 and day 35 due to an open wound at the vaccine injection site. On day 35, all the remaining mice were sacrificed.

#### Detection of anti-RBD antibodies in mouse serum by ELISA

After overnight coagulation at 4°C, blood samples were centrifuged twice at 7000 rpm (10 min, 4°C). Sera were collected and stored at −20°C until further use. Recombinant RBD (GenScript, #Z03483) was used to coat flat-bottom 96-well plates (Thermo Fisher Scientific, #442404) at a final concentration of 2 μg/mL (total Ig plates) or 0.5 μg/mL (IgG1, IgG2a, IgG2b, and IgG3 plates) in PBS for 1 h at 37°C. Plates were washed once with PBS containing 0.1% Tween-20 (PBS-T) and blocked with 1% non-fat dry milk (Santa Cruz, #SC-2325) in PBS-T for 1 h at 37°C. After one wash with PBS-T, serial dilutions (1/100 to 1/72900 in blocking solution) of mouse sera were added and incubated for 45 min at 37°C. A monoclonal antibody against the RBD of SARS-CoV-2 (R&D Systems, 1/500 dilution) was used as a positive control. Plates were washed four times with PBS-T. Biotinylated goat anti-mouse total Ig (Dako, #E0433), IgG1 (Southern Biotech, #1070-08), IgG2a (Southern Biotech, #1080-08), IgG2b (Southern Biotech, #1090-08) and IgG3 (Southern Biotech, #1100-08) antibodies diluted in PBS-T (1/2000) were incubated for 45 min at 37°C. After four washes with PBS-T, plates were incubated with Streptavidin-HRP (Dako, #P0397) diluted in PBS-T (1/2000) for 30 min at 37°C. Plates were washed four times with PBS-T and developed with TMB (Sigma, #T0440) for 10 min. The absorbance was measured on a microplate reader (BioTek Synergy HTX) at 655nm. Actual values were obtained by subtracting blank (wells that were treated like sample wells except adding blocking solution instead of mouse sera) OD values from the actual OD values.

#### Detection of anti-RBM antibodies against wild type, Delta and Omicron strain in mouse serum by ELISA

Three different RBM peptides (aa439 - aa506) of the wild type (Pango lineage B), Delta (Pango lineage B.1.617.2) and Omicron (Pango lineage B.1.1.529) variant were ordered (Proteogenix, France) and reconstituted to a final concentration of 10 mg/mL according to the manufacturer’s instructions. Peptides were coated on flat-bottom 96-well plates (Thermo Fisher Scientific, #442404) at a final concentration of 4 μg/mL overnight at 4°C. The next day, plates were washed once with PBS containing 0.1% Tween-20 (PBS-T) and the protocol described above for the anti-RBD ELISA was followed.

#### Neutralization assay

To measure the neutralizing capacity of induced anti-RBD antibodies, the SARS-CoV-2 surrogate virus neutralization assay from Genscript (USA, #L00847) was performed according to the manufacturer’s instructions. Due to the limited volume of sera derived from mice vaccinated with RBD or CDP-RBD, we were only able to use 1 in 20 serum dilution instead of the recommended 1 in 10 serum dilution.

#### SPR biosensor assay

Surface Plasmon Resonance (SPR) biosensor assays have been carried out using Biacore T200 (GE Healthcare) with CM5 sensor chips (Cytiva). RBD (R&D systems) at a concentration ∼6 μg/mL in 10 mM acetate buffer pH 4.5 was immobilized at the density of ∼2 kRU using the amine-coupling kit (Cytiva) according to the manufacture protocol at the flowrate 5 μL/min. For binding analysis, serum samples were diluted 1:100 in PBS buffer pH 7.4 supplemented with 0.05% Tween-20 and injected over the sensor chip surface at 30 ul/min flowrate, 25°C for 240 s. Dissociation of formed complexes was followed for 180 s after an end of an injection. After each cycle the chip surface was regenerated by 30 s injections of 10 mM Gly, pH 2 and 0.5 M urea.

#### Detection of anti-CDP antibodies in mouse serum by ELISA

CDP was fused to truncated (first 58 C-terminal amino acids) bacterial thioredoxin (uniprot #P0AA25) (TRXtr-CDP). TRXtr-CDP was produced in BL21 (DE3) *E. coli* bacteria (Novagen) as described above and used to coat a flat-bottom 96-well plates (Thermo Fisher Scientific) at a final concentration of 2 μg/mL in PBS for 1 h at 37°C. Plates were washed and blocked as described above. Mouse sera were diluted in 100% BL21 extract, serial dilutions were added and incubated for 45 min at 37°C. For detection of anti-CDP antibodies, a biotinylated goat anti-mouse total Ig (Dako) antibody, diluted in PBS-T (1/2000), was incubated for 45 min at 37°C. Plates were washed four times with PBS-T and they were incubated with Streptavidin-HRP (Dako) diluted in PBS-T (1/2000) for 30 min at 37°C. Plates were further washed, developed and analyzed as described above.

#### Splenocyte isolation and restimulation

Spleens from mice immunized with RBD/MnC or CDP-RBD/MnC were isolated on day 21, cut into small pieces and mechanically dissociated. Splenocytes were passed through a 70 μm cell strainer (Corning, #431751) and spun down for 5 min at 1500 rpm (brake 5, room temperature) in a Rotina 420 R (Hettich) centrifuge. Red blood cells were lysed upon incubation with ammonium-chloride-potassium lysis buffer (150 mM NH_4_Cl, 10 mM KHCO_3_, 100 mM EDTA, pH = 7.4) for 3 min at room temperature. Splenocytes were washed once and then resuspended in RPMI-1640 (Gibco, #L0495-500) medium supplemented with 10% FCS (Biowest, #S0750), 1% Pen/Strep (Gibco, #15140-122), 1% L-glutamine (Brunschwig Chemie BV, #HN08.2) and 50 μM 2-mercaptoethanol (Gibco, #31350-010). Isolated splenocytes were seeded in U-bottom 96-well plates (Greiner Bio-One, #650180) (2∗10^6^ cells/well) and were restimulated *ex vivo* for 5 h with a SARS-CoV-2 peptide mix (Miltenyi Biotec, #130-127-041) in the presence of Brefeldin A (BioLegend, #420604). Peptides of 15 amino acid length with 11 amino acid overlap were used, covering the S1 domain (which contains RBD) of the spike glycoprotein. Unstimulated splenocytes treated with Brefeldin A were taken along as a negative control.

#### Identification of RBD-specific T cells with flow cytometry

After restimulation, cells were transferred to a V-bottom 96-well plate (Greiner Bio-One, #651180) and washed once with PBS. Cells were resuspended in TruStain Fc blocking solution (BioLegend, #101320) for 10 min at room temperature. Afterwards, cells were incubated with anti-mouse CD4-AF700 (BioLegend, #100429, 1/200 dilution), anti-mouse CD8b-AF488 (BioLegend, #126627, 1/200 dilution) and Zombie Aqua fixable viability dye (BioLegend, #423101, 1/200 dilution) diluted in PBS for 20 min on ice. Cells were washed once with PBS and fixed with 4% paraformaldehyde (PFA; Electron Microscopy Sciences, #15710) for 15 min on ice. After fixation, cells were washed once with PBS and permeabilized using the intracellular staining permeabilization wash buffer (BioLegend, #421002) following manufacturer’s instructions. Cell suspensions were then incubated with anti-mouse IFNγ-APC (BioLegend, #505305, 1/200 dilution) and TNFα-PE (BioLegend, #506305, 1/200 dilution) diluted in intracellular staining permeabilization wash buffer for 30 min at room temperature. Cells were washed twice with the permeabilization wash buffer, resuspended in 100 μL PBS and transferred to FACS tubes. Fluorescence intensities and the percentage of IFNγ- and TNFα-expressing cells were measured using an LSRII (BD Biosciences) flow cytometer. Data analysis was performed with the FlowJo V10 software (Tree Star).

#### Hamster housing

Hamsters were housed in type 2 cages with a maximum of two animals per cage under DM(BSL)-II conditions during the acclimatization and vaccination phase. Hamsters were transferred to standard elongated type 2 group cages with two animals per cage under BSL-III conditions (isolators) on the day of virus inoculation using sawdust as bedding with cage enrichment. For all invasive animal procedures, intranasal and intramuscular administration, blood sampling, throat swab collection and euthanasia, the animals were sedated with isoflurane (3–4%/O2). Hamsters were vaccinated according to the schedule via the intramuscular route (i.m) (Figure 4A). Blood was taken via orbital bleeding. Blood samples for serum were immediately transferred to appropriate tubes containing a clot activator. Serum was collected, heat treated, aliquoted and stored frozen.

#### Hamster vaccination

On day 0 and 21 animals were vaccinated with either Tris-sucrose as control or 100 μg CDP-RBD (mixed with Montanide ISA 720 and 67μg CpG oligo 1826, MnC) in a total volume of 100 μL intramuscularly (i.m). Hamsters were injected with a syringe fitted with a 29G (0.33 × 12.7 mm) needle into both hind legs. In short, the hindlimb was extended and inoculum injected with a short fluid movement into the outer thigh (biceps femoris), avoiding the caudal muscles to prevent risk of damage to the sciatic nerve. Animals were placed back in the cage and monitored during recovery.

#### SARS-CoV-2 inoculation

On day 42, all hamsters were challenged with 10^4^ median tissue culture infectious dose (TCID50) SARS-CoV-2 virus particles (BetaCoV/Munich/BavPat1/2020, European Virus Archive Global) intranasal (i.n.) using a dose volume of 100 μL inoculum. On day 4 post challenge half of the animals per group were euthanized by exsanguination under isoflurane anesthesia and necropsy was performed. On day 7 post challenge, the remaining half of the animals per group were euthanized by exsanguination under isoflurane anesthesia and necropsy was performed.

#### Detection of anti-RBD antibodies in hamster serum by ELISA

ELISA was performed as described above. A biotinylated goat anti-hamster IgG antibody (Southern Biotech, #6060-08, 1/2000 dilution) was used to detect hamster antibodies.

#### Sampling post inoculation

Samples from the respiratory tract were collected daily during the challenge phase of the study. In short, throat swabs (FLOQSwabs, COPAN Diagnostic Inc., Italy) were used to sample the pharynx by rubbing the swabs against the back of the animal’s throat saturating the swab with saliva. Subsequently, the swab was placed in a tube containing 1.5 mL virus transport medium (Eagles minimal essential medium containing Hepes buffer, Na bicarbonate solution, L-Glutamin, Penicillin, Streptomycin, BSA fraction V and Amphothericine B), aliquoted in three aliquots and stored.

#### Detection of replication competent virus

Quadruplicate 10-fold serial dilutions were used to determine the virus titers in confluent layers of Vero E6 cells. To this end, serial dilutions of the samples (throat swabs and tissue homogenates) were made and incubated on Vero E6 monolayers for 1 h at 37°C. Vero E6 monolayers were washed and incubated for 5 or 6 days at 37°C. Plates were scored based on the cytopathic effect (CPE) by scoring using the vitality marker WST8. Therefore, WST-8 stock solution was prepared and added to the plates. Per well, 20 μL of this solution (containing 4 μL of the ready-to-use WST-8 solution from the kit and 16 μL infection medium, 1:5 dilution) was added and incubated 3–5 h at room temperature. Subsequently, plates were measured for absorbance at 450 nm (OD_450_) using a micro plate reader and visual results of the positive control CPE were used to set the limits of the WST-8 staining (OD value associated with cpe). Viral titers (log10 TCID50/mL or/g) were calculated using the method of Spearman-Karber.

#### Lung histopathology

Tissue samples (trachea, left lung and left nasal turbinates) were collected, inflated and/or stored in 10% formalin. After fixation, tissues from left lung and left nasal turbinate, gastrointestinal tract were embedded in paraffin. Tissue sections were stained with hematoxylin/eosin for histological examination. Histopathological assessment included aspects like congestion, emphysema, presence of foreign body, haemorrhage, bronchioloalveolar hyperplasia and inflammation and oedema.

### Quantification and statistical analysis

The significance of the difference between experimental groups was evaluated by an unpaired, Mann–Whitney test, unpaired Student’s *t* test or a two-way ANOVA followed by Sidka’s multiple-comparison by Prism (GraphPad) software. A value of p < 0.05 was considered significant.

## Data Availability

Data reported in this paper will be shared by the lead contact upon request. This paper does not report original code. Any additional information required to reanalyze the data reported in this paper is available from the lead contact upon request.
