# Supplementary material for: Vaccination with a bacterial peptide conjugated to SARS-CoV-2 receptor-binding domain accelerates immunity and protects against COVID-19
Source: iScience. 2022 Jul 5;25(8):104719. doi: 10.1016/j.isci.2022.104719 (PMC9252865; doi:10.1016/j.isci.2022.104719)
Supplement: Document S1. Figures S1–S4 [file mmc1.pdf]

iScience, Volume *25*

Supplemental information

Vaccination with a bacterial peptide conjugated to SARS-CoV-2 receptor-binding domain accelerates immunity and protects against COVID-19

Athanasios Blanas, Haiko Karsjens, Aafke de Ligt, Elisabeth J.M. Huijbers, Karlijn van Loon, Stepan S. Denisov, Canan Durukan, Diederik J.M. Engbersen, Jan Groen, Sven Hennig, Tilman M. Hackeng, Judy R. van Beijnum, and Arjan W. Griffioen

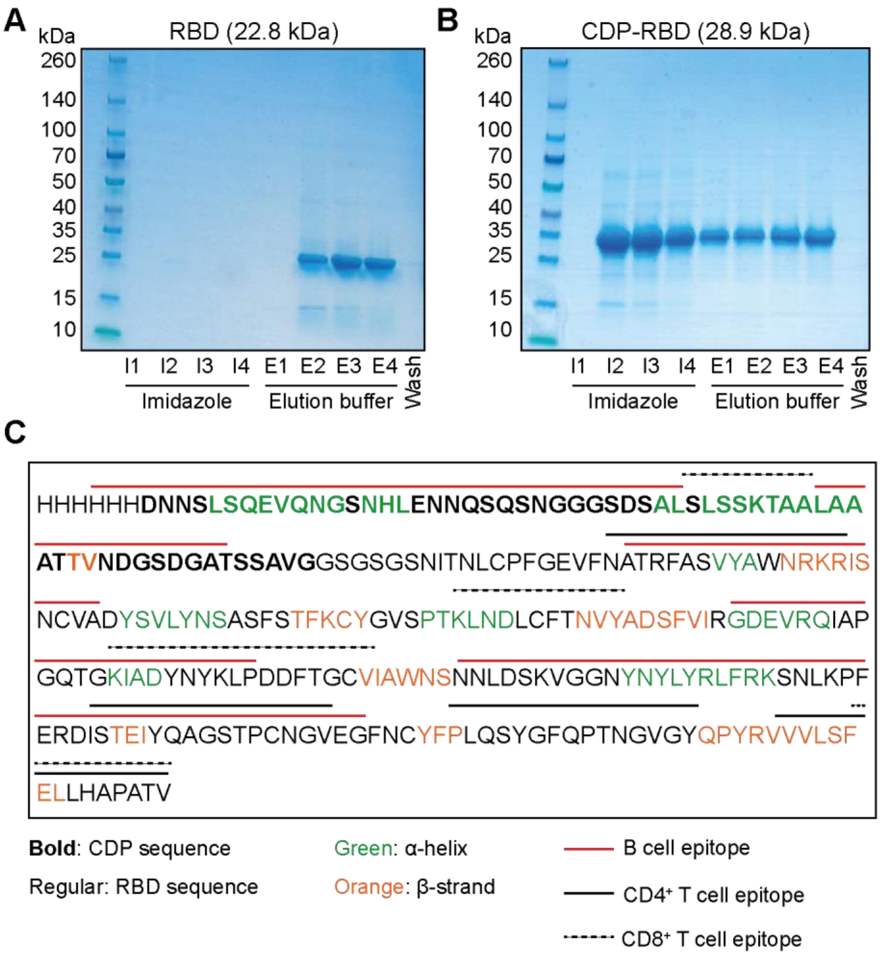

Figure S1: Protein purification and CDP-RBD sequence, related to Figure 1.

(**A, B**) Coomassie staining after SDS-PAGE analysis showing eluted fractions of RBD (**A**) or CDP-RBD (**B**) during purification using Ni-NTA beads. Proteins were eluted with four fractions of 200 mM imidazole, followed by four fractions elution buffer (pH 4.5, 8 M urea). (**C**) Amino acid sequence of the CDP-RBD protein. Predicted secondary structures and B cell as well as T cell epitopes are shown.

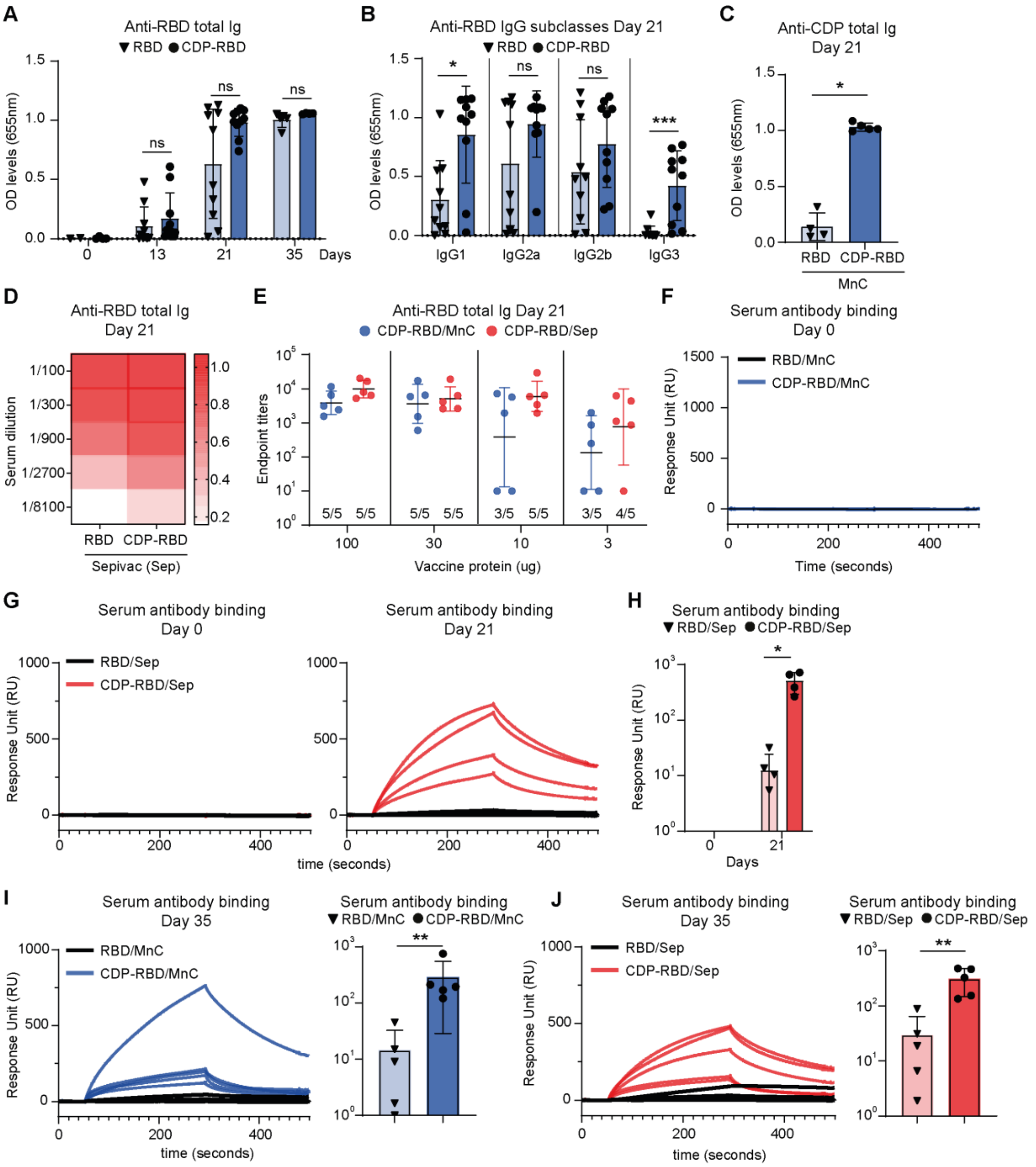

Figure S2: Antibody responses upon vaccination with CDP-RBD, related to Figure 2.

(**A**) Anti-RBD total immunoglobulin (Ig) levels for both vaccine groups at experimental days 0, 13, 21 and 35 were assessed by ELISA. Sera were diluted 1:100. (**B**) Analysis of anti-RBD IgG subclasses at day 21. Mouse sera were diluted 1:100. (**C**) Anti-CDP total Ig levels at day 21. Mouse sera were diluted 1:100. (**D**) Anti-RBD total Ig endpoint titers at day 21 after immunization with RBD or CDP-RBD in combination with the Sepivac adjuvant. (**E**) Effect of different vaccine dosages of CDP-RBD combined with either MnC or Sep on the RBD response. (**F-J**) Surface Plasmon Resonance (SPR) biosensor assay. Binding of serum anti- RBD antibodies derived from mice immunized with RBM/MnC, CDP-RBD/MnC, RBD/Sep and CDP-RBD/Sep to immobilized recombinant RBD protein. Sera were diluted 1:100. Data are shown as mean values ± SD (**A-D, H, I right, J right**) or as geometric mean values ± geometric SD (**E**). Statistical significance was determined by a two-way ANOVA followed by Sidka's multiple-comparison test (**A**) or an unpaired, Mann–Whitney test for each subclass (**B**), vaccine group (**C, I right, J right**), CDP-RBD protein dosage (**E**) or time point (**H**). (ns; no significance, *p<0.05, **p<0.01, ***p<0.001).

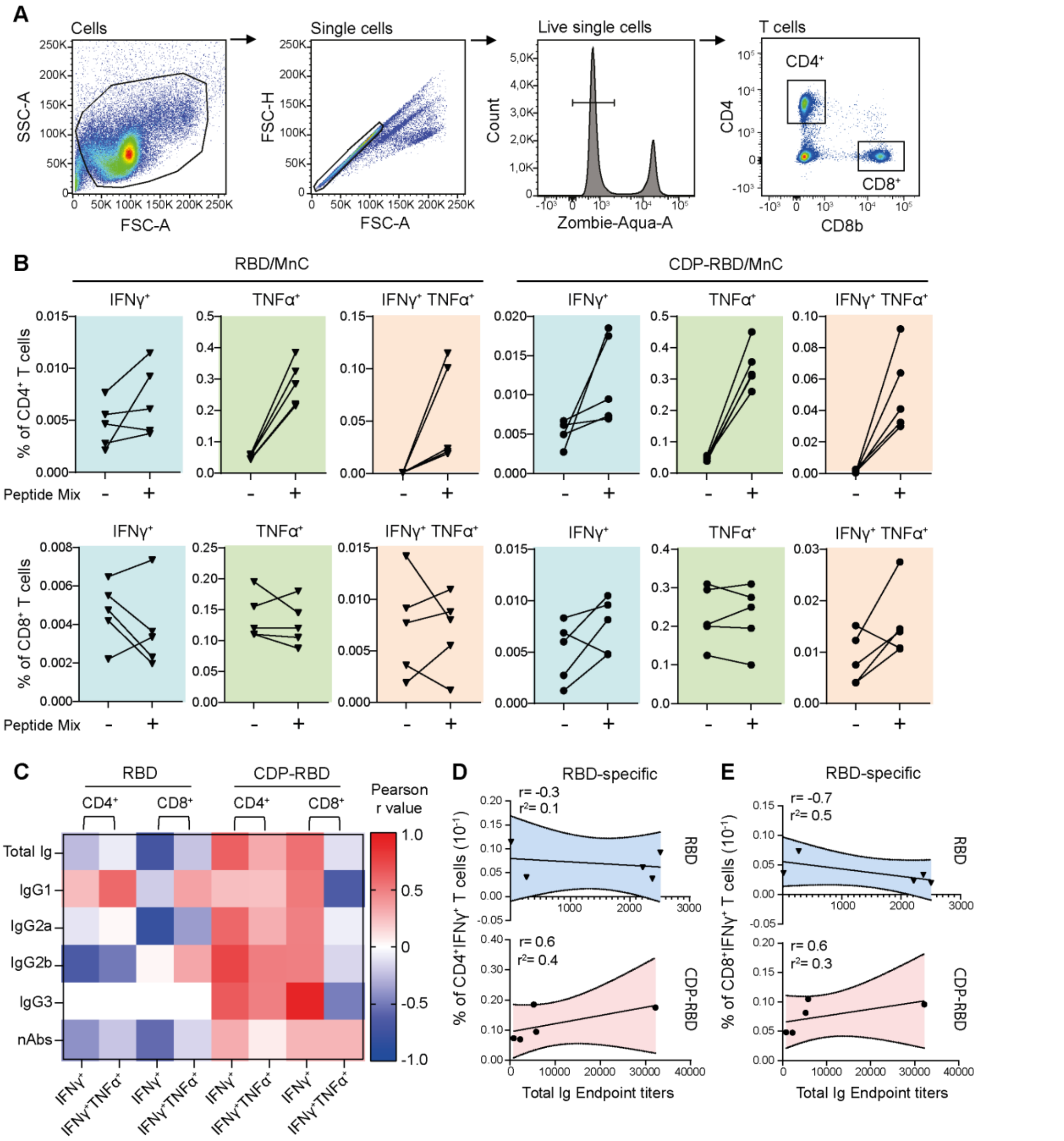

Figure S3: RBD-specific T cell responses induced upon vaccination with RBD/MnC or CDP-RBD/MnC, related to Figure 3.

(**A**) Gating strategy applied during flow cytometric analysis. Gated CD4+ and CD8+ T cells were selected for further analysis. (**B**) Percentage (%) of IFNγ+, TNFα+, and IFNγ+ TNFα+ CD4+ or CD8+ T cells detected in the absence or presence of the SARS-CoV-2 Peptide Mix. Each dot represents the mean value of two technical replicates (n=5 mice per group). (**C**) Heatmap showing the correlation between different components of the humoral and cellular immune response induced in mice 21 days after immunization with RBD/MnC or CDP-RBD/MnC. The Y axis corresponds to antibody end titers (Total Ig, IgG1, IgG2a, IgG2b, IgG3) or the % of RBD:ACE-2 binding inhibition (nAbs), while the X axis corresponds to the % of RBD-specific T cells. The Pearson correlation coefficient (r) value was used for plotting and for estimating the relevance of the features included. (**D, E)** Representative scatter plots illustrating the correlation between the % of RBD-reactive CD4+ (**D**) or CD8+ (**E**) T cells and the corresponding Total Ig endpoint titers. Regression lines denote linear regression for each vaccine group. Both the Pearson correlation coefficient (r) and the coefficient of determination (r2) values are provided for each scatter plot.

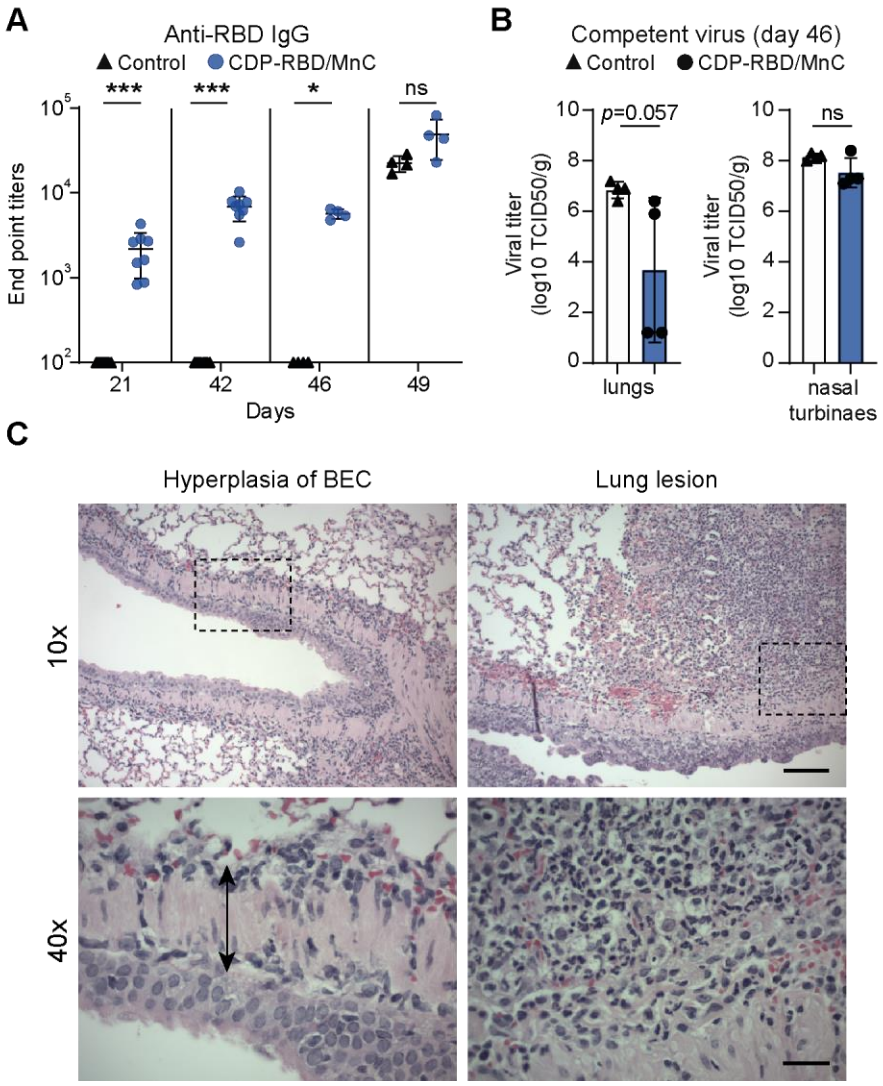

Figure S4: Effects of SARS-CoV-2 inoculation in hamsters, related to Figure 4.

(**A**) Anti-RBD IgG end point titers at experimental days 21, 42, 46 and 49 assessed by ELISA. (**B**) Analysis of competent virus in samples of lung and nasal turbines at experimental day 46. (**C**) HE stained lungs of control animals after SARS-CoV-2 infection. Hyperplasia of bronchial epithelial cells (BEC, double-headed arrow, left) and strong infiltration of immune cells (right) were visible. Scale bars, 100 µm (10x) or 25 µm (40x). Data are shown as geometric mean values ± geometric SD (**A**) or mean values ± SD (**B**). Statistical significance was determined by an unpaired, Mann–Whitney test for each time point (**A**) or organ (**B**).
